# Combination of Multivariate Standard Addition Technique and Deep Kernel Learning Model for Determining Multi-Ion in Hydroponic Nutrient Solution

**DOI:** 10.3390/s20185314

**Published:** 2020-09-17

**Authors:** Vu Ngoc Tuan, Abdul Mateen Khattak, Hui Zhu, Wanlin Gao, Minjuan Wang

**Affiliations:** 1 Key Laboratory of Agricultural Informatization Standardization, Ministry of Agriculture and Rural Affairs, Beijing 100083, China; tuan.vn.nd@gmail.com (V.N.T.); gaowlin@cau.edu.cn (W.G.); 2 College of Information and Electrical Engineering, China Agricultural University, Beijing 100083, China; mateen@aup.edu.pk; 3 Faculty of Electrical and Electronic Engineering, Nam Dinh University of Technology Education, Nam Dinh 420000, Vietnam; 4 Departemnt of Horticulture, The University of Agriculture, Peshawar 25120, Pakistan; 5 Key Laboratory of Liquor Making Biological Technology and Application, Zigong 643000, China; zhuhuiwn@outlook.com; 6 School of Bioengineering, Sichuan University of Science and Engineering, Zigong 643000, China

**Keywords:** ion-selective electrode, multi-ion sensor array, artificial neural network, gaussian process, deep kernel learning, hydroponics

## Abstract

Ion-selective electrodes (ISEs) have recently become the most attractive tools for the development of efficient hydroponic systems. Nevertheless, some inherent shortcomings such as signal drifts, secondary ion interferences, and effected high ionic strength make them difficult to apply in a hydroponic system. To minimize these deficiencies, we combined the multivariate standard addition (MSAM) sampling technique with the deep kernel learning (DKL) model for a six ISEs array to increase the prediction accuracy and precision of eight ions, including $NO_{3}^{-}$, $NH_{4}^{+}$, $K^{+}$, $Ca^{2+}$, $Na^{+}$, $Cl^{-}$, $H_{2}PO_{4}^{-}$, and $Mg^{2+}$. The enhanced data feature based on feature enrichment (FE) of the MSAM technique provided more useful information to DKL for improving the prediction reliability of the available ISE ions and enhanced the detection of unavailable ISE ions (phosphate and magnesium). The results showed that the combined MSAM–feature enrichment (FE)–DKL sensing structure for validating ten real hydroponic samples achieved low root mean square errors (RMSE) of 63.8, 8.3, 29.2, 18.5, 11.8, and 8.8 $\mathrm{mg}\cdot L^{-1}$ with below 8% coefficients of variation (CVs) for predicting nitrate, ammonium, potassium, calcium, sodium, and chloride, respectively. Moreover, the prediction of phosphate and magnesium in the ranges of 5–275 mg·L^−1^ and 10–80 $\mathrm{mg}\cdot L^{-1}$ had RMSEs of 29.6 and 8.7 $\mathrm{mg}\cdot L^{-1}$ respectively. The results prove that the proposed approach can be applied successfully to improve the accuracy and feasibility of ISEs in a closed hydroponic system.

## 1. Introduction

Hydroponics is a modern cultivation system involving aqueous solutions of nutrient salts to grow plants in a soilless culture. This farming system has been deployed widely in modern agriculture, where growers manipulate plant growth by adjusting fertilizer doses to increase crop yield and improve the quality of produce [1,2]. Particularly, the closed hydroponic system has been popularly utilized because of the reusability of the drained solution. The system achieves better efficiency through used water and fertilizer that contributes toward sustainable and environmentally friendly agriculture. However, the closed hydroponic system still poses many challenges such as the imbalance in nutrient ratios caused by the lack or excess of some ions. This is due to a radical flaw in the conventional hydroponic system, where the nutrient concentration is controlled by modulating only electrical conductivity (EC) and pH values [3,4]. To avert this problem, growers normally adopt temporary measures—for example, periodically replacing the nutrient solutions or periodic sampling and analyzing nutrient solutions in a lab. However, these measures are inefficient either due to the use of extra water and fertilizer or loss of nutrient control due to lack of timely feedback. Ion-selective electrodes (ISEs) are considered useful tools to resolve this problem [5]. For example, the array of ISEs determines multi-ions simultaneously in samples of complex hydroponic solutions [6,7,8]. Nevertheless, several difficulties persist with the use of ISEs in the actual hydroponic system due to the effect of interfering ions, ionic strength, temperature noise [9,10], and the drift of ISEs’ output signals [11].

Various approaches have been proposed to resolve the technical challenges of applying ISEs in closed hydroponic systems, such as applying particular sampling [12,13], calibrating techniques [14,15], and compensating interferences supported by machine learning techniques (for example, artificial neural network (ANN) and deep neural network (DNN)) [16,17,18,19,20]. Apart from that, preprocessing techniques as such principle component analysis (PCA) [21], independent component analysis (ICA) [22,23,24], partial least squares (PLS) model [25,26], and the kernel model as Gaussian Process (GP) model, support vector machine model (SVM) [27], etc. have also been used. However, none of technical strategies has radically resolved the drawbacks of ISEs. Other investigations focused on predicting the ions that are not available through an ion-selective electrode (as such $PO_{4}$, $SO_{4}$, and Mg) were examined by fusing the datasets acquired from an array of available ISEs [20,27,28,29].

Previously, ANN was the most attractive approach because it possessed flexibility in processing non-linear problems similar to ISE issues. Good generalization made ANN an essential tool for prediction based on the prior chemical relationships [20]. Nevertheless, the drift of ISEs’ signal was the biggest factor that limited the application of ANN. Recently, ANN has been combined with other models to prepare dataset procedure, such as using the two-point normalization [30]. However, apart from the drift and interferences, ionic strength is a significant factor affecting the accuracy of ISEs [31]. Additionally, the training dataset and calibration sampling procedure considerably affect the performance of the models. Thus, modelers prepared a sufficient calibrating dataset by mixing solutions having individual ions appropriate for the target ranges [17,20]. A hydroponic nutrient solution contains several essential ions with different concentrations ranging from zero to hundreds of $\mathrm{mg}\cdot L^{-1}$ [14,19,32]. This makes the preparation of mixture sample more complicated and time-consuming for acquiring the dataset. Therefore, the ANN does not work satisfactorily with a small dataset of 27 samples [19]. In case of a limited number of samples, GP is an appropriate choice [29]. However, the basic GP model does not fit well in high-dimensional input-output spaces [33]. Recent studies based on the parametric model [22,34] achieved several promising results, for example, simple explicit model construction, minimizing the calibration samples. However, to operate efficiently, these models require other conditions that are not suitable for hydroponic systems. For example, the assumption that the sources are mutually independent [22], the calibration samples for modeling must be in hundreds, and high computational costs [34] making them difficult for application in multi-ion sensing. Wilson et al. [35] proposed the deep kernel learning (DKL) model, which was a combination of DNN and GP models. The model performance was outstanding for image processing [36] and soft sensor purposes [37]. However, DKL was never applied to resolve the problems of ISEs in hydroponics systems. 

**Figure 1:**
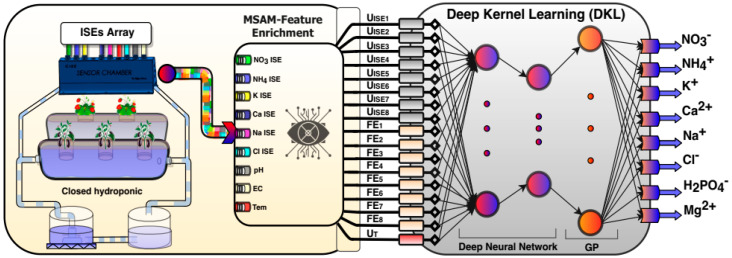
The novel combination of multivariate standard addition (MSAM)–feature enrichment (FE) –deep kernel learning (DKL) architecture for determining multi-ions in hydroponic solutions.

This report presents an approach to combine the multivariate standard addition sampling technique with DKL for solving issues with ISEs to quantify the concentration of eight ions (i.e., nitrate, ammonium, potassium, calcium, sodium, chloride, phosphate, and magnesium) simultaneously in a hydroponic nutrient solution (Figure 1). The performance of DKL was improved by combining ANN (having a good generalizing capability) and GP (having flexibility induced power). This enhanced the accuracy of determining multi-ions in a hydroponic solution by reducing interferences and uncertainties. Moreover, the multivariate standard addition method (MSAM) of sampling [29] was also utilized to prepare the training dataset for DKL. This was to overcome drift, interferences, and ionic strength obstacles of detecting multi-ions in hydroponic solutions. In this approach, we used a deep feed-forward network to extract a high-level representation of the data and also took advantage of the non-parametric flexibility induced by the Gaussian process regression. This improved the predictability of the proposed model for determining ions that are unavailable through commercial ISEs (i.e., phosphate and magnesium ions).

## 2. Materials and Methods

### 2.1. Experiment Preparation 

#### 2.1.1. Sensor Array and Apparatus

**Figure 2:**
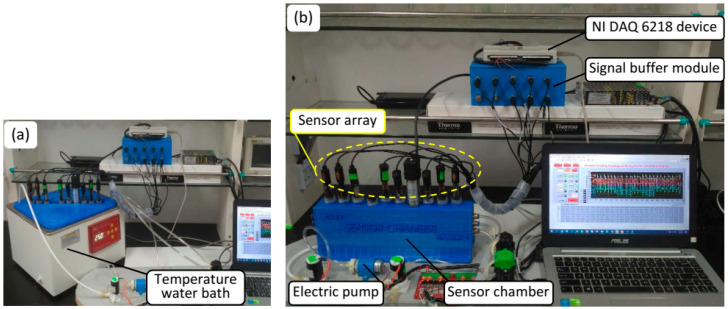
Temperature calibrating water bath (**a**) and the measurement system used in this study (**b**).

In this experiment, a sensor array composed of nine sensors including six commercial ISEs REX 972123, REX 972122, pNa 701, pCl 202 (Shanghai INESA, Shanghai, China), Orion 9719BNWP, and Orion 9320BNWP (Thermo Fisher Scientific, Waltham, MA, USA) for determining six ions (i.e., nitrate, ammonium, sodium, chloride, potassium, and calcium) and three transducers including an electrical conductivity probe (DJS-1C; Shanghai INESA, Shanghai, China), a pH electrode (E-201F; Shanghai INESA, Shanghai, China), and a temperature probe (Pt100; Yuace, Shanghai, China) for detecting conductivity, pH, and temperature of the samples was deployed. The basic specifications of the sensors are summarized in Table 1. The suite of sensors was plugged into a sensor chamber made of acrylonitrile butadiene styrene (ABS) plastic by a 3D printer connected with an electric pump (KLP05-6, Kamoer, Shanghai, China) to create a simple flow injection sampling structure. The sensor chamber was immersed into a temperature calibration water bath HH.S21-4 (Boxun, Shanghai, China) for calibrating interfered temperature, as illustrated in Figure 2a. Finally, the sensors were connected to a signal buffer module based on INA116 (Texas Instruments, Dallas, TX, USA) and a data acquisition device NI USB DAQ 6218 (National Instrument Corporation, Austin, TX, USA). The data were collected by lab program based on LabVIEW (National Instrument Corporation, Austin, TX, USA) and then processed by the proposed models to enhance the accuracy of ISEs. The connected system is depicted in Figure 2a,b.

#### 2.1.2. Sampling Preparation

In this study, data samples for developing the models were prepared from standard solutions in the lab for training and testing purposes. The samples were collected from real hydroponic systems for validating the model performance. To mimic the complex interaction among the ions of the actual hydroponic solution, one hundred training samples ($100\;=\;10^{2}$, for 10 levels of six factors i.e., six ions, as shown in Table 2) were prepared using the fractional factorial design technique [25,38]. Furthermore, the samples at different levels ($27\;=\;3^{3}$, $36\;=\;6^{2}$, and $64\;=\;8^{2}$ samples, corresponding with three, six, and eight levels of the six considered factors) were also used to estimate the efficiency of the number of levels for the performances of the models. Additionally, two elements, dihydrogen phosphate and magnesium, were also studied by randomly changing the concentration. A water bath was used to adjust the temperature of samples randomly from 15 °C to 35 °C, corresponding with the temperature of the actual hydroponic system, to supply information for neutralizing the temperature interference. 

The MSAM sampling method [29] was utilized to prepare the dataset for developing the models. Deionized water was mixed with a sufficient amount of stock solution of targeted ions to dilute the rinsed water (RW) in which the concentration of each ion species approximated to the lower limit value ($C_{0}$) of linearity range of the ISEs (roughly 1 $\mathrm{mg}\cdot L^{-1}$). In order to imitate the real conditions for the training samples, a base solution (BS) was prepared by mixing the Hoagland standard solution [39] and tap water (1/1 “*v*/*v*”). A standard solution (SS) of 40 mL was prepared by mixing the appropriate amounts of the BS and the stock solutions of potassium nitrate, potassium chloride, potassium dihydrogen phosphate, magnesium sulfate, ammonium nitrate, ammonium dihydrogen phosphate, calcium nitrate, calcium chloride, sodium chloride, and sodium nitrate. This way, the concentration of the considered ions was set from the first level to the tenth level of 44–1328, 6–120, 15–500, 10–350, 5–300 and 5–350 $\mathrm{mg}\cdot L^{-1}$ for $NO_{3}^{-}$, $NH_{4}^{+}$, $K^{+}$, $Ca^{2+}$, $Na^{+}$, and $Cl^{-}$, respectively (Table 2). A random increase in range of 6–678 and 6–125 $\mathrm{mg}\cdot L^{-1}$ for $H_{2}PO_{4}^{-}$ and $Mg^{2+}$, respectively, was achieved. In the sampling procedure, first, 80 mL RW was injected into the sensor chamber. Then, the electric pump retained the solution flow to the ISEs’ surface until the stabilization of their electromotive force (EMF) values ($U_{0}$) (about 1 min). Subsequently, the SS was injected into the chamber and cycled for 3–5 min (depending upon the concentration of the sample) until the potentials ($U_{x}$) of the electrodes were stable. The stabilized EMF values were acquired by a lab program based on LabVIEW (LabVIEW 2017, National Instrument Corporation, Austin, TX, USA), and the measured values were exported to a comma-separated-values (CSV) file as the dataset to develop the models (Figure 2b). All of the models were developed by Python 3.6.2, Scikit-Learn library, SciPy, and several third-party libraries. 

To evaluate the feasibility of the proposed models, 10 actual hydroponic nutrient solution samples were collected from various hydroponic systems developed for nine plant species (lettuce, perilla, purple bok choy, Chinese cabbage, strawberry, Gynura bicolor DC, amaranth, eggplant, and tomato) in the College of Information and Electrical Engineering, China Agricultural University, Beijing, China (CIEE, CAU), as shown in Table 3. The actual concentration values of the collected samples were analyzed at the Laboratory of Agricultural Informatization Standardization, Ministry of Agriculture and Rural Affairs, CAU, Beijing, China, and at the International Joint Research Center of Aerospace Biotechnology and Medical Engineering, Beihang University, Beijing, China. Furthermore, the direct calibration method (in which the array of sensors was directly immersed into the measured solutions, and the responding EMFs were recorded by sampling program) was also carried out for comparison with the proposed approach.

### 2.2. Development of Models for Determining Multi-Ion

#### 2.2.1. Neural Network Model

An artificial neural network (ANN) is a powerful and widely used algorithms [40]. In this study, a deep feed-forward network following the standard forward propagation and back-propagation was used to develop the regression model.

**Figure 3:**
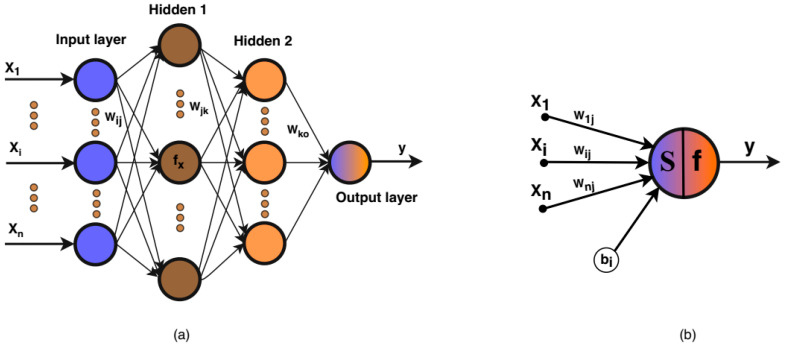
Neural network structure diagram (**a**) and the neuron (**b**).

Figure 3 illustrates the simple structure of an ANN and the neuron [41]. An ANN has an input layer, which consists of $x_{i}\;$(*i* = 1, 2, …, *p*) independent variables (the signals from sensors), L layers of hidden *k* (*k* = 1, 2, …, *S*) number of neurons for each, and $y_{i}$ outputs. In the feed-forward stage, the input $x_{i}$ connects to each single input node, and that node transmits a weight value $w_{ij}$ and a bias $b_{i}$ to the hidden layer. Subsequently, the sum of these input-weight products is passed through an activation function $f(z_{i})$ to determine how to change the outcome. Equation (1) is the mathematical expression of the $i^{th}$ neuron,

(1) $$y_{i}=f\left(z_{i}\right)=f\left(\sum_{i}w_{ij}x_{i}+b_{i}\right)$$

where $x_{i}$ is the input information of neuron *i*, $w_{i}$ is the network connection weight,$\;f\left(z_{i}\right)$ is the activation function, $b_{i}$ is the bias, and $y_{i}$ is the output value.

The normally used activations include the sigmoid function $f(z)\;=\;\frac{1}{{1+e}^{-z}}$, the Tanh function $f\left(z\right)\;=\;\frac{2}{{1+e}^{-2z}}-\;1$, and the ReLU function $f\left(z\right)\;=\;\{\begin{array}{c} 0,\;z<0\\ z,\;z\geq 0 \end{array}$, etc. 

At the back-propagation stage, the weights and bias parameters of the networks are learned by incrementally adjusting the produced values of the network approaches to the expected values from the training data. The gradient-descent algorithm is a regular optimization method. Suppose a cost function C, expression of the weights (W), the biases (B), the single input ($X_{i}$), the single modeled output (${\hat{Y}}_{i}$), actual output ($Y_{i}$), i.e., $C\left(W,\;B,\;X_{i},\;{\hat{Y}}_{i},\;Y_{i}\right)$, represents the difference between the model’s predicted outputs and the actual outputs. The derivatives of C corresponding to each weight and bias value in each layer of the network could be determined using the chain rule, such as

(2) $$\frac{\partial C}{\partial w_{ij}^{\left(L\right)}}=\frac{\partial C}{\partial z_{j}^{\left(L\right)}}\cdot \frac{\partial z_{j}^{\left(L\right)}}{\partial w_{ij}^{\left(L\right)}}$$

(3) $$\frac{\partial C}{\partial b_{j}^{\left(L\right)}}=\frac{\partial C}{\partial z_{j}^{\left(L\right)}}\cdot \frac{\partial z_{j}^{\left(L\right)}}{\partial b_{j}^{\left(L\right)}}$$

Then, the weights and biases are updated by stochastic gradient descent optimization method,

(4) $$w_{ij}\leftarrow \;w_{ij}-\gamma \frac{\partial C}{\partial w_{ij}^{\left(L\right)}}$$

(5) $$b_{j}\leftarrow \;b_{j}-\gamma \frac{\partial C}{\partial b_{j}^{\left(L\right)}}$$

where γ is the learning rate.

#### 2.2.2. Gaussian Process Model

A Gaussian process (GP) is a stochastic process f(x) characterized by its mean function μ(x) and covariance function $k\left(x,x^{\prime }\right)$ [42]. In the Gaussian process regression task, consider a set of data D consisting of N input vectors $X\;=\;x_{1},\;x_{2},\;\ldots \;x_{N}$ (signals of dimension D from sensors) is a multivariate Gaussian distribution with corresponding continuous outputs $y\;=\;y_{1},\;y_{2},\;\ldots \;y_{N}$ (ion concentration of samples). In a Gaussian process regression (GPR) model, an output is assumed to be noisily observed from an underlying functional mapping f(x). Then with the set of data $D\;=\;\left\{X^{\left[i\right]},y^{\left[i\right]}\right\}$:

(6) $$y^{\left[i\right]}=f\left(x^{\left[i\right]}\right)+\varepsilon^{\left[i\right]}$$

where *i* = 1, 2, … *n*, $\varepsilon \;=\;N\left(0,\sigma_{f}^{2}\right)$ is the additive independent Gaussian noise with mean 0 and variance $\sigma_{f}^{2}$. The collection of function values f will have a joint Gaussian distribution if f(x) is a GP.

(7) $$f\left(X\right)={\left[f\left(x_{1}\right),f\left(x_{2}\right),\ldots ,f\left(x_{n}\right)\right]}^{T}\sim N\left(\mu ,\Sigma \right)$$

where mean vector $\mu \;=\;\mu \left(x_{i}\right)$ and covariance matrix $\Sigma \;=\;k\left(x_{i},x_{j}\right)$, determined by the mean function and kernel function of the GP.

Normally, the mean function of the GP is assumed to be zero. Thus, the relation from one to the other is only the covariance function $k\left(x,x^{\prime }\right)$. A popular kernel function is the radial basic function (RBF; also called squared exponential), given as follows:

(8) $$k\left(x,\overset{´}{x}\right)=exp\left[\frac{-{\left(x-x^{\prime }\right)}^{2}}{2l^{2}}\right]$$

where *l* is length-scale, which quantifies the level of local smoothness level of the drawn Gaussian process distribution. 

Consider another dataset $D_{*}\;=\;\left\{x_{*}^{\left[i\right]},y_{*}^{\left[i\right]}\right\}$, $i\;=\;1,\;2,\ldots ,\;n_{*},$ which refers to the testing set, has the same distribution as the set D, then defined by the Gaussian process [42], we have

(9) $$\left[\begin{array}{c} f\left(X\right)\\ f\left(X_{*}\right) \end{array}\right]\sim N\left(0,\;\left[\begin{array}{cc} K\left(X,X\right) & K\left(X,X_{*}\right)\\ K\left(X_{*},X\right) & K\left(X_{*},X_{*}\right) \end{array}\right]\right)$$

where K(X,X), $K\left(X_{*},X_{*}\right)$, and $K\left(X_{*},X\right)$ are covariance matrices evaluated at training locations X, testing locations $X_{*}$, and locations $X_{*}\;\mathrm{and}\;X$, respectively.. Then, given the additive Gaussian noises, and using the rules for conditioning Gaussian distributions, the posterior distribution of the GP is [42]

(10) $$f\left(X_{*}\right)|y(X)\sim N\left(\mu_{*},\Sigma_{*}\right)$$

where the posterior mean $\mu_{*}\;=\;m\left(X_{*}\right)+K\left(X_{*},X\right){\left[K\left(X,X\right)+\sigma^{2}I\right]}^{-1}\left(y\left(X\right)-m\left(X\right)\right)$ and the posterior variance $\Sigma_{*}\;=\;K\left(X_{*},X_{*}\right)-K\left(X_{*},X\right){\left[K\left(X,X\right)+\sigma^{2}I\right]}^{-1}K\left(X,X_{*}\right)$, $m\left(X_{*}\right)$ and m(X) are mean vectors calculated at $X_{*}$ and X, I is the identity matrix.

The predictive distribution of the GP is based on Equation (10). The kernel learning algorism is carried out by maximizing the log marginal likelihood of the targets y. The probability of the data conditioned only on kernel parameters θ is given as

(11) $$logp(Y|\theta ,X)\propto -Y^{T}(K_{\theta }(X,X)+\sigma^{2}I{)}^{-1}Y-log|K_{\theta }(X,X)+\sigma^{2}I|$$

#### 2.2.3. Deep Kernel Learning Model

Recently, deep kernel learning (DKL), a combination of the deep learning structure and kernel methods, was proposed as an elegant and flexible algorithm [35]. In this study, the multi-ion concentration of the hydroponic nutrient solution was determined by combining the MSAM technique [29] with DKL signal processing to enhance the accuracies and feasibilities of the ISEs, as shown in Figure 1. The collected signals from sensor array extracted more significant information by using the feature enrichment technique. In this manner, eight signals from six ISEs, pH, and EC probe were enriched to 16 data features composing of eight original features and eight feature enrichment (FE) data. The principle of the technique is illustrated by MSAM feature enrichment component in Figure 1 and Equation (12).

(12) $$U_{FEij}=U_{xij}-U_{0ij}$$

where *i* is the number of samples (1–100), j is the number of ion-selective electrodes (1–8), and $U_{0ij}$, $U_{xij}$ are the potential of corresponding ISE at concentration $C_{0}$ and $C_{x}$ respectively. $U_{FEij}$, “enriched data value,” represents the values of differences between $U_{0ij}$ and $U_{xij}$, which were used to improve the performance of the model. The details of this technique were reported by Tuan et al. [29]. The 17-dimensional input vector (16 enriched features plus one temperature sensor) was then introduced to the DKL, as shown in Figure 1. 

The data of the DKL model were first propagated by the neural network in the forward stage. The high-dimensional MSAM-FE data (17 dimensions) were transformed into the lower-dimensional feature vector, which was suitable for the input arguments of Gaussian process regression. The expected values of the concerned ions were predicted by DKL, relying on the posterior distribution as a function of the input data. A Gaussian process is equivalent to a Bayesian neural network that has an infinite number of nodes [43]. Therefore, the ended Gaussian process layer of the DKL structure could be considered as an infinite number of nodes hidden in the deep neural network layer. This architecture greatly increases the expression ability of the network compared to a stand-alone deep neural network. Nevertheless, the Gaussian processes naturally do not fit well with the high-dimensional input–output spaces. The additive deep neural network acts as a feature extractor and dimensionality reduction method, which compensates more robustly for the Gaussian process regression.

The DKL could be viewed as a Gaussian process with a stand-alone deep kernel [35]. Therefore, the DKL could be constructed from a base kernel $k(x^{\left[i\right]},x^{\left[j\right]}|\theta )$ with kernel parameters θ, as follows:

(13) $$k(x^{\left[i\right]},x^{\left[j\right]}|\theta )\to k(g\left(x^{\left[i\right]},w\right),g\left(x^{\left[j\right]},w\right)⎣\theta ,w)$$

where *g*(*x*, *w*) is a non-linear mapping performed by the neural network, w is weight parameter. The kernel is the core of DKL in this study. The DKL model was conducted with the RBF, Dotproduct, and the spectral mixture kernel [35], as per Wilson and Adams [44].

(14) $$k_{SM}\left(x,x^{\prime }|\theta \right)\;=\;\sum_{q\;=\;1}^{Q}a_{q}\frac{{\left|\Sigma_{q}\right|}^{\frac{1}{2}}}{{\left(2\pi \right)}^{\frac{D}{2}}}\exp\left(-\frac{1}{2}\|\sum_{q}^{\frac{1}{2}}\left(x-x^{\prime }\right){\|}^{2}\right)cos\langle x-x^{\prime },2\pi \mu_{q}\rangle$$

where the learnable kernel parameters $\theta \;=\;\left\{a_{q},{\;\Sigma }_{q},\mu_{q}\right\}$ include a weight, an inverse length scale, and a frequency vector for each of the Q spectral components.

Constructing the DKL involves optimizing learnable parameters including network weights and kernel parameters and also tuning hyper-parameters such as the learning rate, number of iterations, and number of nodes (neurons) in each layer of the neural network. Before training could be carried out, suitable values of the hyper-parameters were specified. We executed this based on cross-validation over a small hyper-parameter search-space and thus employed a systematic optimization-based procedure. In this manner, we determined the major hyper-parameters, such as the number of nodes in each hidden layer, the prior white-noise level of the Gaussian process, and the number of epochs, i.e., training iterations and the learning rate. The parameters that were included in the model are listed in Table 4. 

The parameters of the deep kernel learning (including neural network parameters *w*, and Gaussian process kernel parameter θ) were learned by maximizing the log posterior marginal likelihood applying Equation (11), with respect to γ = {w,θ}. The derivatives concerning the weight variables $\frac{\partial_{g}\left(x,w\right)}{\partial w}$ were computed using the standard back-propagation algorithm. To avoid local minima and overfitting, the dropout regularization algorithm was used for training the network [45] with the adjusted dropout rate of 0.5 to 0.99.

#### 2.2.4. Model Performance Metrics

To evaluate the efficiency of the proposed models for determining multi-ion concentration in hydroponic solution, three performance indices were estimated, i.e., the performance coefficient $\left(R^{2}\right)$, the root mean square error (RMSE), and the coefficient of variation (CV). The smaller the values of RMSE, the closer the predicted values are to the true values, which means better prediction accuracy. The closer the $R^{2}$ value to unity, the better the machine learning prediction is. Moreover, the CV represents the precision of the model performance. CV is negatively correlated with the accuracy of predicted values. The RMSE is given as follows:

(15) $$RMSE\;=\;\sqrt{\frac{1}{n}\sum_{i=1}^{n}{\left(y_{i}-{\hat{y}}_{i}\right)}^{2}}$$

where *n* is the total number of data in the training set or test set, $y_{i}$ is the actual ion concentration value, and ${\hat{y}}_{i}$ is the predicted ion concentration value.

$R^{2}$ is an index that measures the degree of agreement between the test data and the fitting function. It is presented as

(16) $$R^{2}\;=\;1-\frac{\sum_{i}{\left(y_{i}-{\hat{y}}_{i}\right)}^{2}}{\sum_{i}{\left(\bar{y_{i}}-{\hat{y}}_{i}\right)}^{2}}$$

where ${\bar{y}}_{i}$ is the average of the test set.

The CV is evaluated by the following expression:

(17) $$CV\;=\;\frac{SD}{{\bar{y}}_{N}}\cdot 100\;=\;\frac{\sqrt{\frac{\sum_{i}^{N}{\left({\hat{y}}_{i}-{\bar{y}}_{s}\right)}^{2}}{N-1}}}{{\bar{y}}_{N}}\cdot 100$$

where SD is the standard deviation, the average concentration estimated by the model for each sample ${\bar{y}}_{s}$, the average concentration of N measurements ${\bar{y}}_{N}$, and N is the number of sample measurements.

## 3. Results

### 3.1. Responses of the Ion Selective Electrodes

As mentioned above, the ISEs were calibrated by two methods: the direct calibration method (DCM) [46], and the multivariate standard additional method (MSAM). The Nikolsky–Eisenman calibration equation concerning the logarithmic relationship between the concentrations of the standard samples (x) and EMFs of electrodes (y) is summarized in Table 5. The results showed that the determination coefficient ($R^{2}$) of the ISEs using the DCM was 0.90 to 0.95. In this MSAM-based approach, the $R^{2}$ significantly improved from 0.92 to 0.97. However, these achieved improvements still did not fulfill the requirements of multi-ion measurement in the actual hydroponic solution. Therefore, the models were deployed to improve the efficiency of electrodes for quantifying ions (nitrate, ammonium, potassium, calcium, sodium, and chloride) simultaneously. Furthermore, the models also fused the data collected from available ISEs to predict two ions (phosphate and magnesium), which had no feasible ISEs.

### 3.2. Determination of the DKL Architecture 

The structure of DKL developed with the main network parameters and hyper-parameters (Table 4) was subjected to various trials to determine the best fit. The best DKL model architecture consisted of a five-layer neural network and a Gaussian process with RBF kernel. The Tanh and ReLU activation functions were applied to construct the hidden layer of the ANN stage. To avoid local minima and overfitting problems, the dropout algorithm was used for training the network. We adopted the standard root mean squared error (RMSE) loss function and the Adam optimizer for tuning the model [47]. Furthermore, to evaluate the most effective model for determining the concentrations of multiple ion species in the hydroponic nutrient solution, the ANN (having the same architecture as the ANN stage of the DKL) and the GP models were tested as well. The fitted DKL architecture was configured as in Table 6.

**Figure 4:**
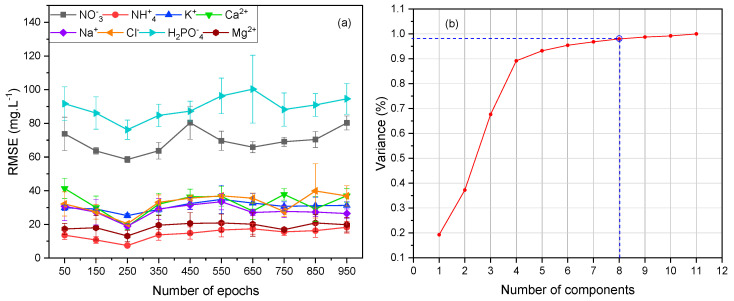
Relationship between the number of epochs and RMSEs (**a**) and PC components and variance of the dataset (**b**).

The prediction RMSEs of the DKL, according to the number of epochs, are illustrated in Figure 4a. The DKL was converged at 250 epochs, as most of the ion predictions achieved the lowest errors RMSEs. The number of nodes of the last hidden layer was determined by relying upon the number of considered targets (target ions) and the coefficient of principal component (PC) analyzed from the high dimensional data of the MSAM-FE. Choosing a suitable degree of PC from the raw data of ISEs eliminates the noise [48], and improves the efficiency of GP performance. The ratio of PCs and variance of data is shown in Figure 4b. The first eight PCs contained roughly 98% of data variances. As per a previous study, there are about 3% system errors and noise in the ISEs based measurement system [49]. Therefore, the number of PCs could be chosen as eight and the last three PCs removed whilst losing roughly 2% of data variances.

### 3.3. Evaluation of the Performance of Proposed Models

**Figure 5:**
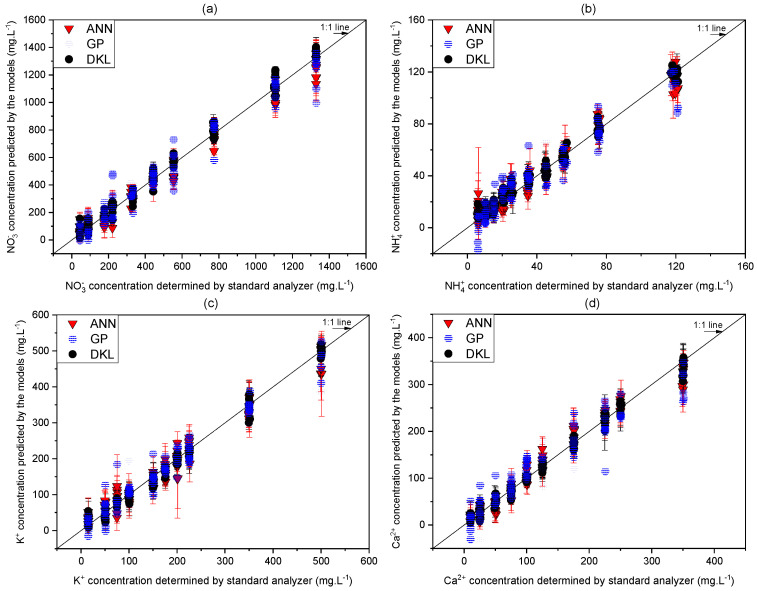

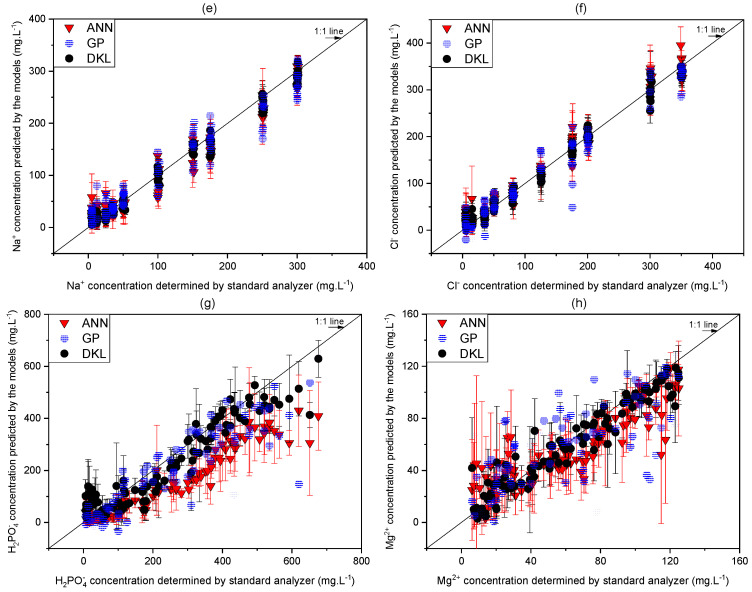
Relationships between predicted ion concentrations of the models and standard analyzers. (**a**) $NO_{3}^{-}$, (**b**) $NH_{4}^{+}$, (**c**) $K^{+}$, (**d**) $Ca^{2+}$, (**e**) $Na^{+}$, (**f**) $Cl^{-}$, (**g**) $H_{2}PO_{4}^{-}$, and (**h**) $Mg^{2+}$. Error bars indicate standard deviations of three replicates.

In this work, three models were conducted using Python language and several supported libraries. Normalized data allows the model to learn rapidly. Therefore, the raw dataset was preprocessed first in the range of −1 to 1 using MinMaxScaler of the Scikit Learn library. In the training stage, the k-fold cross-validation algorithm (k = 10) was used to evaluate RMSE and $R^{2}$ based on the metrics to find out the fitted model for determining the multi-ion. The performance results of the three models are presented in Figure 5. The predicted nitrate is shown in Figure 5a. The results of the ANN and the GP showed a relatively linear and accurate prediction with $R^{2}$ of 0.95 and 0.94, a slope of 0.95 and 0.94, and the RMSEs of 91.5 mg·L^−1^ and 102.7 $\mathrm{mg}\cdot L^{-1}$, respectively. In the DKL case, the results were better than those of ANN and GP with RMSE of 58.5 $\mathrm{mg}\cdot L^{-1}$. Specifically, the highly linear relationship (having an $R^{2}$ of 0.98 and a slope of 1.01) revealed that the training of the DKL was achieved well.

For the ammonium prediction (Figure 5b), the performance of ANN and GP were slightly low (RMSE of 10.9 $\mathrm{mg}\cdot L^{-1}$ and 13.1 $\mathrm{mg}\cdot L^{-1},$ $R^{2}$ of 0.92 and 0.90, and slope of 0.92 and 0.90 for ANN and GP, respectively). Although the performance of DKL was better than those of the ANN and GP models, it just archived with an RMSE of 7.4 $\mathrm{mg}\cdot L^{-1},$ an $R^{2}$, and a slope of 0.95. The imperfect prediction results of ammonium might be caused by strong interferences of other ions in the hydroponic solution samples. 

The GP model was effective for the prediction of potassium (Figure 5c) with an RMSE of 31.2 $\mathrm{mg}\cdot L^{-1},$ an $R^{2}$ of 0.96, and a slope of 0.95, providing a better performance than that of the ANN model. The DKL model exhibited the best performance with an RMSE of 25.2 $\mathrm{mg}\cdot L^{-1},$ a slope of 0.99, and an $R^{2}$ of 0.978.

Calcium prediction was more stable and linear for ANN than for GP (Figure 5d). Specifically, the GP showed a slightly low linear relationship (RMSE 35.3 $\mathrm{mg}\cdot L^{-1}$, $R^{2}$ 0.92, and slope 0.85), which was relatively lower than those of the ANN (RSME 23.6$\;\mathrm{mg}\cdot L^{-1}$, $R^{2}$ 0.96, and slope 0.98) and the DKL (RMSE 18.8 $\mathrm{mg}\cdot L^{-1}$, $R^{2}$ 0.97, and slope 0.99) models.

The sodium and chloride prediction results (Figure 5e,f) showed similar trends in terms of RMSEs, $R^{2}$, and slopes. The performance of the DKL was the best, with the RMSE of 18.9 and 20.3 $\mathrm{mg}\cdot L^{-1}$, the $R^{2}$ of 0.96 and 0.97, and the slope of 0.97 and 0.99 for sodium and chloride, respectively. In general, most of the $R^{2}$ and slopes of the DKL model were relatively high (0.95 to 0.99), which showed that the training stage of the models was at the acceptable levels [50].

The phosphate and magnesium prediction results (Figure 5g,h) showed that the ANN performance was slightly preferable to GP. However, neither of the models provided expected results. The RMSEs of ANN and GP models were 122.5 mg·L^−1^ and 135.8 $\mathrm{mg}\cdot L^{-1}$, respectively, for phosphate and 21.3 mg·L^−1^ and 25.2$\;\mathrm{mg}\cdot L^{-1}$, respectively, for magnesium. Moreover, unstable results were observed for both models. Conversely, the DKL provided relatively satisfying result, exhibiting 76.2$\;\mathrm{mg}\cdot L^{-1}$ RMSE, 0.86 $R^{2}$ and 0.85 slope for phosphate and 13.1 $\mathrm{mg}\cdot L^{-1}$ RMSE, 0.89 $R^{2},$ and 0.88 slope for magnesium. The details of correlation values of the actual concentrations versus the predicted concentrations of the models are summarized in Table 7.

**Figure 6:**
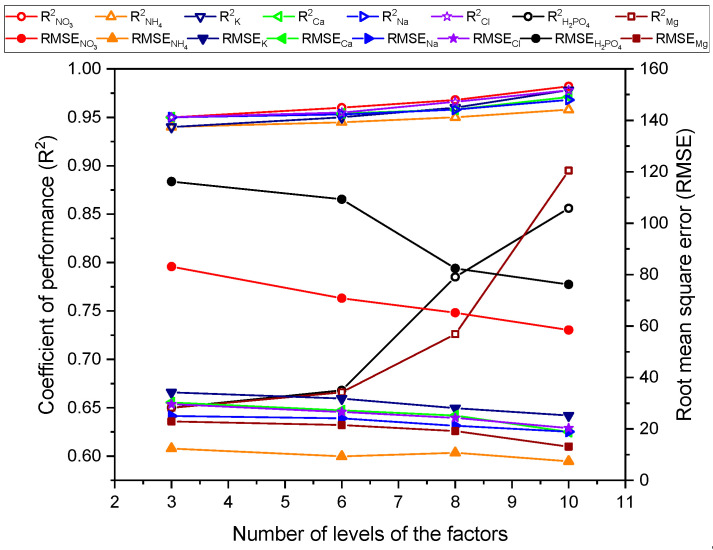
The performance and efficiency of the DKL model with varying number of levels of factors.

Furthermore, the DKL was also trained with three smaller datasets (27, 36, and 64 samples corresponding to 3, 6, and 8 levels of six factors, respectively) to estimate which sample size was appropriate. The results (Figure 6) showed that the ISEs prediction of ions was slightly improved having the RMSEs reduced from 5 $\mathrm{mg}\cdot L^{-1}$ (minimum) to 25.6 $\mathrm{mg}\cdot L^{-1}$ (maximum), and the gain in $R^{2}$ was from 0.018 to 0.032 for ammonium and nitrate, respectively. Particularly, the predictions of two unavailable ISEs ions had considerable improvement at the tenth level. The RMSEs reduced to 40.1 mg·L^−1^ and 9.8 $\mathrm{mg}\cdot L^{-1}$, and the $R^{2}$ increased to 0.205 and 0.245 for determining phosphate and magnesium, respectively.

### 3.4. Validation of the Proposed Models with Real Hydroponic Samples

**Figure 7:**
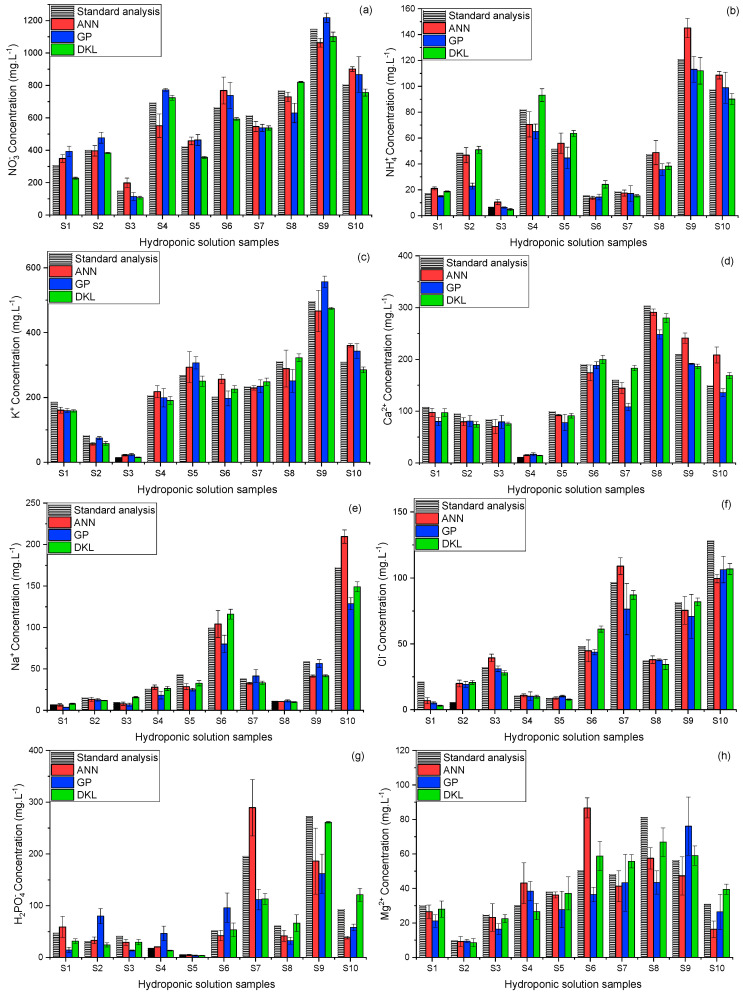
Comparison of the actual concentrations with the predicted concentrations determined by proposed models using ten different hydroponic samples (**a**) $NO_{3}^{-}$, (**b**) $NH_{4}^{+}$, (**c**) $K^{+}$, (**d**) $Ca^{2+}$, (**e**) $Na^{+}$, (**f**) $Cl^{-}$, (**g**) $H_{2}PO_{4}^{-}$, and (**h**) $Mg^{2+}$. Error bars indicate standard deviations of three replicates.

After training and cross-validating with the laboratory-made samples, the applicability of the models for determining multi-ion was validated with real hydroponic samples. The relationship of the ion concentration predicted by the models and the standard analyzers is shown in Figure 7. In most sample measurements, the ANN predictions were more accurate than those of the GP. The ion concentrations predicted by the DKL model were closer to the actual concentrations than those of ANN and GP, which indicated that the DKL model improved the accuracy of the sensor array by processing the signals effectively.

Table 8 summarizes the RMSEs obtained from the three models. In potassium prediction, the RMSE of GP (35.2$\;\mathrm{mg}\cdot L^{-1}$) was lower than that of the ANN (36.3$\;\mathrm{mg}\cdot L^{-1}$). In most of the cases, the DKL model achieved the best predictability with the smallest RMSEs and CVs below 8%. In the prediction of phosphate and magnesium, even though the error bars showed relatively high CVs (13.9% and 14.8%) for DKL method, the prediction results were almost comparable to the actual values. This implies that the DKL model could be potentially deployed for developing multi-ion sensor for sensing phosphate and magnesium in hydroponic nutrient solutions.

## 4. Discussion

We proposed a combination of the multivariate standard addition sampling technique and three machine learning models to develop an architecture for effectively determining the concentrations of multiple ion species in a hydroponic nutrient solution. The aim was to compensate the potential drifts, interferences from secondary ions and temperature, and ionic strength problems of the ISEs. 

The results of the training stage (Figure 5 and Table 7) showed that the GP-based structure resolved most of the fundamental problems of ISEs array with relatively high linear relationship ($R^{2}$ = 0.96) and low RMSE (31.2$\;\mathrm{mg}\cdot L^{-1}$) for potassium prediction. The positive results with GP may be attributed to the exceptional processing ability of the GP in the scarce dataset (limited dataset) [51]. However, for predicting other ions (ammonium, calcium, and sodium), the GP responses were not adequate, although the MSAM technique had eliminated some issues of ISEs, as shown in Table 5 (Section 3.1). These unsatisfactory results may be due to the interferences of other ions that affected the Nernstian slope [16,52]. Another concern was the inherent weakness of GP [53] faced with the complex high dimensional input-output (i.e., 17-dimensional input signal, and eight outputs) of the multi-ion sensing in the hydroponic system. The ANN model was a suitable model to overcome interference problems. This was demonstrated by the predicted results of ANN, which were better than those of GP in case of several ions (Table 7). Having the ability of non-linear processing and high effective generalization, ANN may resolve the interferences [17,19]. Nevertheless, in coping with complex problems such as multi-ion interferences of the hydroponic solution, the ANN had some shortcomings that were not completely rectified. The ANN predicted results of most ions (Figure 5 and Table 7) were comparable with those of previous studies [19]. However, the ANN responses were still slightly unstable and had moderate error bar levels. Therefore, the previous studies normally utilized ANN to overcome one pair of interference of ions [17,54] or some ions [19]. To process the high dimensional data efficiently, the models need to be fed with the dataset having hundreds of samples or more [55] to obtain accurate predictions. For determining multi-ion (eight ions) simultaneously, it is difficult to prepare the large dataset. However, small datasets with 27 samples, for example [19], may not be appropriate because the high number of dimensions and small size of the dataset compromises the robustness of ANN. This limitation motivated researchers to innovate by combining ANN with other techniques, e.g., PCA-ANN, ICA-ANN [48] to improve its efficiency. Thus, the DKL was acquired to solve the problems of the ISEs based sensing system. As shown in Figure 5 and Table 7, the results of DKL were better than those of ANN and GP models in all the cases. The model provided the highest relationship ($R^{2}$ = 0.98), slope (1.01), and the lowest RMSE of 58.5, 7.4, 25.2, 18.8, 18.9, 20.3, 76.2, and 13.1 $\mathrm{mg}\cdot L^{-1}$ for predicting nitrate, ammonium, potassium, calcium, sodium, chloride, phosphate, and magnesium, respectively. 

In the real hydroponic samples test, the prediction results of the models (Figure 7 and Table 8) tended to emulate those of the training stage. The results of the GP model in potassium prediction (35.2 $\mathrm{mg}\cdot L^{-1}$ RMSE, and 8.8% CV) were better than those of ANN. However, in most of the remaining cases, the ANN predictions were better than those of GP. For a detailed comparison, the RMSE results of both models were higher than those of the DKL model. This may be due to the differences in background ions and the drifts of ISEs’ signals. However, the differences were rather small, and the CVs of most cases were less than 10%. It proves that the MSAM is a simple and effective sampling technique for multi-ion sensing in the hydroponic system that could considerably restrict the drifts, changing background ions, and ionic strength effects. The best results of the DKL model in both real hydroponic solution and training test may be created by compensating the advantages of both ANN and GP together [35]. In this manner, the role ANN stage in DKL was analogous with a simple data mining and preprocessing stage. The high-dimensional dataset of multi-ion sensor array was reduced by the non-linear projection of ANN. The number of nodes in the last hidden layer was chosen following the relationship between the components and variance of the data (as shown in Figure 4b) and the number of output targets for dimensional reduction purposes. The reduced-dimension dataset was then introduced to the last GP stage of DKL to mitigate uncertainties and allow for accurate predictions. The fitted parameters and hyper-parameters of two halves of DKL structure revealed the robustness of DKL for better prediction results with CVs below 8% and the lowest RMSE of 63.8, 8.3, 29.2, 18.5, 11.8, and 8.8 $\mathrm{mg}\cdot L^{-1},$ for the prediction of nitrate, ammonium, potassium, calcium, sodium, and chloride, respectively.

In phosphate and magnesium predictions, the generalized and expressive abilities of the DKL model produced significant differences in results compared with those of both ANN and GP models. The DKL model achieved RMSE of 29.6 mg·L^−1^ and 8.7 $\mathrm{mg}\cdot L^{-1}$ and CVs below 15% for prediction of phosphate and magnesium, respectively (see Table 8). Additionally, even with the smallest number of samples (27 samples), the prediction results of the DKL model were relatively consistent with the actual concentrations (Figure 7). The results show that the MSAM sampling and the feature enrichment technique provided more useful information to models, which would improve the efficiency of prediction and inferences. Moreover, the results proved that the DKL model successfully fused the data of ISEs to retrieve the concentrations of the two unavailable ISEs elements. The predicted effects of phosphate and magnesium were not as high as those of other available ISEs ions. Nevertheless, in conditions where sensors are lacking, the proposed approach could be used as a foundation to develop phosphate and magnesium diagnosing tool for the closed hydroponic system.

## 5. Conclusions

This study proposed a combination of the MSAM-FE technique and the DKL model for developing a sensing architecture that could prevent the adverse effects on ISEs, such as signal drifts, interferences, and the ionic strength for reliably determining eight ions in the hydroponic nutrient solution. The parameters and hyper-parameters of the models were trained by 100 imitated hydroponic background samples and validated by 10 samples collected from several real hydroponic systems. 

The results showed that the ANN and GP models did not accomplish high satisfaction as expected because of the effects of interferences, even though they were supported by the MSAM sampling technique for minimizing drifts and ionic strength. The combined MSAM-FE-DKL model enhanced the reliability of the multi-ion sensing with the RMSE of 63.8, 8.3, 29.2, 18.5, 11.8, 8.8, 29.6, and 8.7 $\mathrm{mg}\cdot L^{-1}$ and the CVs below 8% for predictions of nitrate, ammonium, potassium, calcium, sodium, chloride, phosphate, and magnesium, respectively. Specifically, in phosphate and magnesium predictions, the DKL-based structure exhibited many desirable outcomes, and the results were akin to those of the actual ISEs. These proved that the MSAM-FE-DKL sensing architecture can be used as a soft phosphate and magnesium sensing tool for the multi-ion testing tasks.

The proposed approach enabled us to quantify eight essential ions simultaneously in hydroponic solution with satisfying results and a feasible structure, suggesting that the proposed multi-ion sensing architecture could be applied for improving the quality and efficiency of closed hydroponic systems. The study also paved the way for effectively measuring and controlling individual ion tasks in a hydroponic system. Although DKL provided several promising results, it still has some limitations, such as computational complexity and storage of GP core [56]. This limitation may make DKL difficult to deploy in the big data system. However, in this study, this problem was alleviated by reducing the dimension of data (from seventeen to eight) and utilizing the finite samples (one hundred). Thus, the computational burden hinders no more with the application of DKL in this scenario. Moreover, the DKL model was combined with a relatively simple sampling technique. This approach has feasible application in the closed hydroponic system. Nevertheless, deploying MSAM-FE-DKL in commercial hydroponics needs further research to validate the efficiency of DKL via developing fertigation through the hydroponic solution. In future, DKL could be combined with semi-supervised or unsupervised learning techniques for effectively adopting ISEs into commercial hydroponic systems.

## Figures and Tables

**Table 1:** Characteristics of the ion-selective electrodes (ISEs) and other sensors used in the study.

Sensor	Measurement Range	Membrane Type	Response Time (s)	Manufacturer
Nitrate ISE: REX972123	0.6–60000 $\mathrm{mg}\cdot L^{-1}$	PVC	~50	Shanghai INESA, China
Ammonium ISE: REX 972,122	0.02–14000 $\mathrm{mg}\cdot L^{-1}$	PVC	~50	Shanghai INESA, China
Potassium ISE: Orion 9719BNWP	0.04–39000 $\mathrm{mg}\cdot L^{-1}$	PVC	~50	Thermo Fisher, USA
Calcium ISE: Orion 9720BNWP	0.02–40000 $\mathrm{mg}\cdot L^{-1}$	PVC	~50	Thermo Fisher, USA
Sodium ISE: pNa 701	0.03–23000 $\mathrm{mg}\cdot L^{-1}$	Glass	~50	Shanghai INESA, China
Chloride ISE: pCl 202	0.35–3500 $\mathrm{mg}\cdot L^{-1}$	PVC	~50	Shanghai INESA, China
pH electrode: E-201F	2–14	-	~50	Shanghai INESA, China
EC electrode: DJS-1C	0–10000 μS	-	-	Shanghai INESA, China
Temperature probe: Pt100	0–100 ℃	-	-	Yuace, China

**Table 2:** The ranges concentration of considered ions prepared training samples.

Ions	Level 1	Level 2	Level 3	Level 4	Level 5	Level 6	Level 7	Level 8	Level 9	Level 10
Nitrate ($\mathrm{mg}\cdot L^{-1}$)	44	88	177	221	332	442	553	769	1106	1328
Ammonium ($\mathrm{mg}\cdot L^{-1}$)	6	10	15	20	25	35	45	55	75	120
Potassium ($\mathrm{mg}\cdot L^{-1}$)	15	50	75	100	150	175	200	225	350	500
Calcium ($\mathrm{mg}\cdot L^{-1}$)	10	25	50	75	100	125	175	225	250	350
Sodium ($\mathrm{mg}\cdot L^{-1}$)	5	12	25	35	50	100	150	175	250	300
Chloride ($\mathrm{mg}\cdot L^{-1}$)	5	15	35	50	80	125	175	200	300	350

**Table 3:** The real hydroponic solution samples used to validate the models.

Sample	Grown Plant	Growing Period	Nutrient Standard	Sampling Sites
**S1**	Lettuce 1	Three weeks	Hoagland’s solution	Experimental Plant Factory, CIEE, CAU
**S2**	Perilla	Five weeks	Hoagland’s solution	Experimental Plant Factory, CIEE, CAU
**S3**	Lettuce 2	Four weeks	Hoagland’s solution	Experimental Plant Factory, CIEE, CAU
**S4**	Purple bok choy	Five weeks	Hoagland’s solution	Experimental Plant Factory, CIEE, CAU
**S5**	Chinese cabbage	Six weeks	Yamazaki’s solution	Experimental Plant Factory, CIEE, CAU
**S6**	Strawberry	Eight weeks	Hoagland’s solution	Experimental Plant Factory, CIEE, CAU
**S7**	Gynura bicolor DC	Five weeks	Hoagland’s solution	Experimental Plant Factory, CIEE, CAU
**S8**	Amaranth	Four weeks	Yamazaki’s solution	Experimental farm, CIEE, CAU
**S9**	Eggplant	Twelve weeks	Yamazaki’s solution	Experimental farm, CIEE, CAU
**S10**	Tomato	Six weeks	Yamazaki’s solution	Experimental farm, CIEE, CAU

**Table 4:** The structure of the DKL for predicting multi-ion concentration.

**Parameters**	**Values**
Number of hidden layers	1, 2, 3, 4, 5, 6
Hidden layer size	1 to 1000
Hidden layer transfer function f(x)	tansig, logsig, linear, ReLU
Output layer transfer function	ReLU
Optimization algorithm	Stochastic gradient descent (SGD), Broyden–Fletcher–Goldfarb–Shanno (BFGS), Adam
Dropout rate	0.5 to 0.99
Learning rate	0.001 to 0.1
Max number of epochs	1000
Prior whitenoise level	0.001 to 1
Kernel	Radial basic function (RBF), Dotproduct, Spectral mixture (SM)
Training goal	10^−6^

**Table 5:** The responses of the ISEs calibrated by the direct calibration method (DCM) and MSAM technique.

ISEs	DCM		MSAM	
	$R^{2}$	Calibrating Equation	$R^{2}$	Calibrating Equation
Nitrate	0.93	y = −22.04ln(x) + 202.62	0.95	y = −22.86ln(x) + 208.47
Ammonium	0.90	y = 22.856ln(x) − 255.01	0.92	y = 22.92ln(x) − 253.87
Potassium	0.95	y = 23.39ln(x) − 240.07	0.97	y = 23.07ln(x) − 237.03
Calcium	0.94	y = 11.419ln(x) − 79.31	0.96	y = 11.06ln(x) − 76.90
Sodium	0.91	y = 18.227ln(x) − 178.31	0.93	y = 19.76ln(x) − 186.22
Chloride	0.94	y = −23.28ln(x) + 186.54	0.96	y = −23.02ln(x) + 192.33

**Table 6:** The structures of the fitted DKL model for predicting multi-ion concentration.

Layer 1			Layer 2			Layer 3			Layer 4			Layer 5			Opt	LR	N.o.E	KF
N.o.N	AF	DR	N.o.N	AF	DR	N.o.N	AF	DR	N.o.N	AF	DR	N.o.N	AF	DR	Adam	0.005	250	RBF
580	Tanh	0.99	580	Tanh	0.99	100	ReLU	0.99	100	ReLU	0.99	8	ReLU	0.99				

N.o.N: umber of nodes, AF: activation function, DR: dropout rate, Opt: optimizer, LR: learning rate, N.o.E: number of epochs, KF: kernel function.

**Table 7:** The correlation of the predicted concentrations (y) with the actual values (x).

Species	Models	Predicting Equation	RMSE $\left(\mathrm{mg}\cdot L^{-1}\right)$	Coefficient of Performance ($R^{2}$)
**Nitrate**	**ANN**	y = 0.95x + 18.11	91.5	0.95
	**GP**	y = 0.94x − 10.93	102.7	0.94
	**DKL**	y = 1.01x + 17.81	58.5	0.98
**Ammonium**	**ANN**	y = 0.92x + 4.54	10.9	0.92
	**GP**	y = 0.90x + 3.80	13.1	0.90
	**DKL**	y = 0.95x + 5.13	7.4	0.95
**Potassium**	**ANN**	y = 0.94x + 7.33	33.5	0.95
	**GP**	y = 0.95x + 11.50	31.2	0.96
	**DKL**	y = 0.99x + 5.59	25.2	0.978
**Calcium**	**ANN**	y = 0.98x + 4.20	23.6	0.96
	**GP**	y = 0.85x + 22.30	35.3	0.92
	**DKL**	y = 0.99x + 5.27	18.8	0.97
**Sodium**	**ANN**	y = 0.94x − 1.97	22.5	0.94
	**GP**	y = 0.86x + 14.11	29.3	0.92
	**DKL**	y = 0.97x + 1.08	18.9	0.96
**Chloride**	**ANN**	y = 0.97x + 1.22	25.0	0.95
	**GP**	y = 0.95x + 5.67	27.2	0.94
	**DKL**	y = 0.99x + 2.68	20.3	0.97
**Phosphate**	**ANN**	y = 0.71x + 31.59	122.5	0.76
	**GP**	y = 0.62x + 20.27	135.8	0.61
	**DKL**	y = 0.85x + 17.82	76.2	0.86
**Magnesium**	**ANN**	y = 0.82x + 11.43	21.3	0.75
	**GP**	y = 0.63x + 10.64	25.2	0.62
	**DKL**	y = 0.88x + 2.11	13.1	0.89

**Table 8:** Comparison of the predicted quality of the proposed models with the real hydroponic solution tests.

Considered Ions	Range of Concentration $(\mathrm{mg}\cdot L^{-1}$)	Models	Accuracy (RMSE, $\mathrm{mg}\cdot L^{-1}$)	Precision (CV, %)
**Nitrate**	150–1150	ANN	83.8	7.2
		GP	86.1	8.4
		DKL	63.8	3.5
**Ammonium**	6–120	ANN	10.3	9.2
		GP	12.2	10.3
		DKL	8.3	7.0
**Potassium**	15–500	ANN	36.3	9.2
		GP	35.2	8.8
		DKL	29.2	5.4
**Calcium**	10–305	ANN	25.2	7.7
		GP	29.1	9.6
		DKL	18.5	5.5
**Sodium**	6–175	ANN	14.8	9.2
		GP	17.1	9.9
		DKL	11.8	6.8
**Chloride**	1.6–128	ANN	11.7	9.5
		GP	12.8	10.5
		DKL	8.8	6.9
**Phosphate**	5–275	ANN	50.5	22.3
		GP	55.8	23.6
		DKL	29.6	13.9
**Magnesium**	10–80	ANN	16.9	21.3
		GP	18.1	23.5
		DKL	8.7	14.8
